# Glucose treatment of human pancreatic β-cells enhances translation of mRNAs involved in energetics and insulin secretion

**DOI:** 10.1016/j.jbc.2021.100839

**Published:** 2021-05-27

**Authors:** Albatoul Zakaria, Claire Berthault, Bertrand Cosson, Vincent Jung, Ida Chiara Guerrera, Latif Rachdi, Raphael Scharfmann

**Affiliations:** 1 Institut Cochin, INSERM U1016, CNRS UMR 8104, Université de Paris, Paris, France; 2 Epigenetics and Cell Fate Center, CNRS UMR 7216, Université de Paris, Paris, France; 3 Plateforme protéomique Necker, INSERM US24/CNRS UMS3633, Université de Paris, Structure Fédérative de Recherche Necker, Paris, France

**Keywords:** human beta cell (β-cell), translation regulation, glucose metabolism, insulin, tricarboxylic acid cycle (TCA cycle/Krebs cycle), 4EBP1, 4E-binding protein 1, eIF, eukaryotic initiation factor, FACS, fluorescence-activated cell sorting, MAPK, mitogen-activated protein kinase, mTOR, mammalian target of rapamycin, PCSK1, proprotein convertase subtilisin/kexin type 1, PKA, protein kinase A, PRKAR2A, protein kinase type II-alpha regulatory subunit 1, RAPA, rapamycin, rpS6, ribosomal protein S6, SUnSET, surface sensing of translation, TCA, tricarboxylic acid

## Abstract

Glucose-mediated signaling regulates the expression of a limited number of genes in human pancreatic β-cells at the transcriptional level. However, it is unclear whether glucose plays a role in posttranscriptional RNA processing or translational control of gene expression. Here, we asked whether glucose affects posttranscriptional steps and regulates protein synthesis in human β-cell lines. We first showed the involvement of the mTOR pathway in glucose-related signaling. We also used the surface sensing of translation technique, based on puromycin incorporation into newly translated proteins, to demonstrate that glucose treatment increased protein translation. Among the list of glucose-induced proteins, we identified the proconvertase PCSK1, an enzyme involved in the proteolytic conversion of proinsulin to insulin, whose translation was induced within minutes following glucose treatment. We finally performed global proteomic analysis by mass spectrometry to characterize newly translated proteins upon glucose treatment. We found enrichment in proteins involved in translation, glycolysis, TCA metabolism, and insulin secretion. Taken together, our study demonstrates that, although glucose minorly affects gene transcription in human β-cells, it plays a major role at the translational level.

In insulin-producing pancreatic β-cells, glucose uptake tightly regulates both protein expression levels and cellular function. However, chronic high glucose weakens pancreatic β-cell response, leading to the loss of β-cell function, which is a main issue of type 2 diabetes mellitus (1, 2). The critical role of glucose in insulin secretion is well described both in rodent and human β-cells (3). Its role in protein biosynthesis, both at the transcriptional and translational levels, has also been deeply studied in rodent β-cells (4, 5, 6). As examples, exposure of the rat β-cell line INS-1E to high glucose induced the expression of more than 2000 genes within 12 h (4). Moreover, in rat islets, glucose coordinately increased the translation of proinsulin, and the proconvertase PC1/3 (also named Pcsk1), an endopeptidase that is necessary for the conversion of proinsulin into insulin (7).

Less information is available on whether and how glucose regulates protein expression in human β-cells. Recently, using the human β-cell line EndoC-βH1 (8) and human islets, we unexpectedly observed through global transcriptomic analyses that glucose regulates an extremely limited number of genes (9). This suggested that glucose might control protein expression levels through translational regulation.

To study glucose-regulated translation in human β-cells, we adapted the surface sensing of translation (SUnSET) technique in order to capture and identify the nascent proteome. The SUnSET technique was developed by Schmidt *et al.* (10), allowing the monitoring and quantification of global protein translation by Western blotting, fluorescence activated cell sorting (FACS), or immunohistochemistry. This is a nonradioactive technique, based on a pulse of puromycin that is followed by the use of anti-puromycin antibodies to detect newly puromycin-labeled peptides. Puromycin is an aminonucleoside antibiotic and has a structural similarity to aminoacyl-transfer RNA (11). Briefly, puromycin reaction requires its incorporation into growing peptide chains by the formation of a peptide linkage. The result is the formation of a puromycin-tagged nascent peptide chain unable to undergo further elongation (11). Of interest, at the low concentration used in SUnSET, puromycin binds only at the C terminus of the full-length protein, allowing one to monitor full-length protein translation (12). SUnSET is now validated as an accurate alternative to traditional isotope methods for measuring relative rates of protein synthesis in cells (10, 13, 14, 15, 16, 17, 18).

Here, we describe and validate the use of the SUnSET approach to demonstrate through FACS and Western blot that glucose increases protein translation in the human cell line EndoC-βH2 (19). We next defined and quantified newly synthesized proteins by mass spectrometry. We identified more than 2000 proteins using this approach. We demonstrate that, although glucose regulates a very limited number of genes in human β-cells (9), it activates the expression of a large number of proteins (more than 700) involved in the translation machinery, in glucose metabolism and in insulin exocytosis. Among these proteins, we observed that, in human β-cells, glucose increased the translation of proprotein convertase subtilisin/kexin type 1 (PCSK1) without any effect on PCSK2, another member of the same proconvertase family.

Taken together, we validate the use of the SUnSET approach in human β-cells and demonstrate that glucose regulates the human β-cell proteome through specific protein translation.

## Results

### Glucose induces mTOR signaling in EndoC-βH2 cells

**Figure 1:**
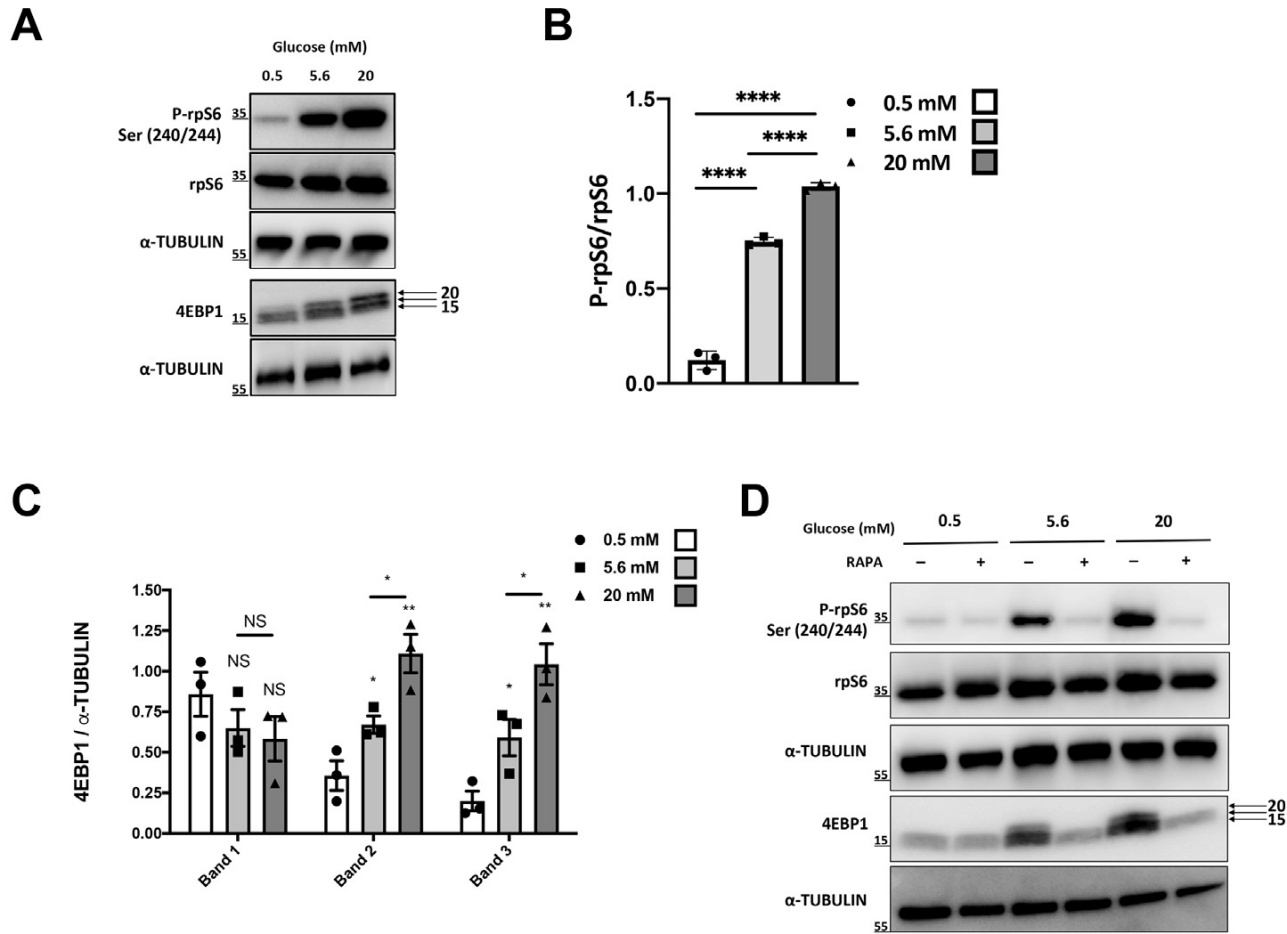
**Glucose regulates mammalian target of rapamycin activity in EndoC-βH2 cells.** EndoC-βH2 cells were glucose starved for 24 h and next pulsed with different concentrations of glucose for 30 min. Proteins were prepared and analyzed by Western blot. *A*, detection of P-rpS6, rpS6, and 4EBP1. α-TUBULIN was used as a loading control. *B*, quantification of P-rpS6 phosphorylation relative to total rpS6. *C*, quantification of 4E-BP phosphorylation relative to total α-TUBULIN. The error bars represent the mean ± SEM of three separate experiments. NS, not significant, ∗*p* < 0.05, ∗∗*p* < 0.01, ∗∗∗∗*p* < 0.001. *D*, EndoC-βH2 cells were treated without or with rapamycin (RAPA: 50 nM) for 1 h before glucose pulses. Detection of P-rpS6, rpS6, and 4EBP1 is represented. α-TUBULIN was used as a loading control.

Glucose plays major roles in β-cells, not only for insulin secretion but also to tightly regulate protein levels (6, 20). Unexpectedly, we previously observed by a transcriptomic analysis that glucose modulates the expression of an extremely limited number of genes in the human β-cell line EndoC-βH1 (9). Hence, we hypothesized that glucose regulation in β-cells might mainly take place at a posttranscriptional level, affecting protein translation. Activation of the mammalian target of rapamycin (mTOR) pathway is tightly linked to protein translation (21), and its activation is mainly associated to amino acid availability (22). Of interest, glucose is also a potent activator of the mTOR pathway in β-cells (23, 24). Therefore, we first tested whether glucose stimulates mTOR activity in the human β-cells EndoC-βH2. Following 24-h starvation, glucose treatment (30 min) induced a concentration-dependent increase in the phosphorylation of ribosomal protein S6 (P-rpS6), a downstream target of mTOR complex1 (mTORC1/S6 kinase pathway) at serine 240/244 (Fig. 1, *A* and *B* for quantification). A parallel increase was also observed for the phosphorylation of eukaryotic translation initiation factor 4E-binding protein 1 (4EBP1), another downstream target of mTOR signaling (Fig. 1, *A* and *C* for quantification).The phosphorylation of rpS6 and 4EBP1 by glucose was blocked by rapamycin (RAPA), a specific inhibitor of mTORC1 (Fig. 1*D* and Fig. S1, *A* and *B* for quantification). Taken together, glucose activates the mTOR pathway in EndoC-βH2 cells, which suggests that it regulates protein translation.

### Glucose upregulates protein translation in EndoC-βH2 cells

**Figure 2:**
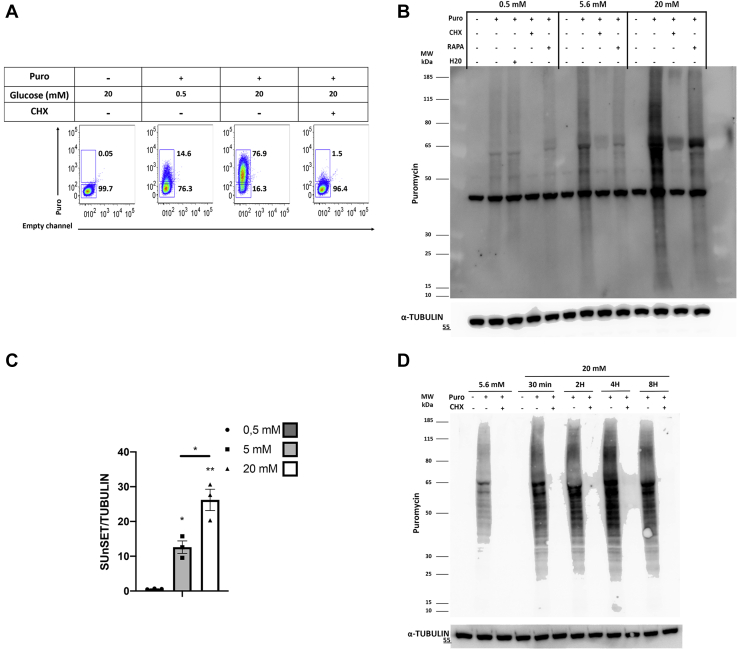
**Glucose increases protein translation in EndoC-βH2 cells as measured by puromycin incorporation.** EndoC-βH2 cells were glucose starved at 0.5 mM for 24 h. Glucose was next added at the indicated concentrations for 30 min and puromycin (10 μg/ml) for the last 10 min. In some cases, EndoC-βH2 cells were treated for 1 h with either RAPA (50 nM) or cycloheximide (CHX; 50 μg/ml) before glucose pulse. *A*, detection of puromycin incorporation by FACS (representative of two independent experiments). *B*, detection of puromycin incorporation by Western blot. *C*, quantification of puromycin incorporation relative to α-TUBULIN. The error bars represent the mean ± SEM of three separate experiments. ∗*p* < 0.05, ∗∗*p* < 0.01. *D*, EndoC-βH2 cells were cultured at 5.6 mM glucose. Glucose concentration was next increased to 20 mM for 30 min, 2 h, 4 h, and 8 h, with puromycin added during the last 10 min. Cycloheximide (CHX) was used as described above. Puromycin incorporation was detected by Western blot.

To study the effect of glucose on protein translation in EndoC-βH2 cells, we used the SUnSET method that is based on the quantification of the rate of puromycin incorporation into newly synthesized proteins (10). For this purpose, we glucose-starved EndoC-βH2 cells for 24 h, followed by a 30-min glucose pulse (20 mM) and the addition of puromycin during the last 10 min before cell collection. There, we first analyzed the effect of glucose on puromycin incorporation into EndoC-βH2 cells using FACS (Fig. 2*A*). We observed that 20 mM glucose increased puromycin incorporation, which was blocked when EndoC-βH2 cells were pretreated with cycloheximide, a protein synthesis inhibitor (Fig. 2*A*). We next analyzed puromycin incorporation by Western blot at 0.5, 5.6, and 20 mM glucose. Under these settings, glucose treatment increased puromycin incorporation in a glucose-dependent manner (Fig. 2, *B* and *C* for quantification). Of importance, glucose did not increase puromycin incorporation in EndoC-βH2 cells when the cells were pretreated with cycloheximide. Moreover, rapamycin pretreatment sharply decreased puromycin incorporation upon glucose stimulation (Fig. 2*B*). Taken together, glucose stimulated protein synthesis in EndoC-βH2 cells in an mTOR-dependent fashion. We next performed a time course of glucose effect on puromycin incorporation. We cultured EndoC-βH2 cells at 5.6 mM glucose and pulsed them with 20 mM glucose for 30 min, 2 h, 4 h, and 8 h with puromycin added during the last 10 min. Western blot analyses indicated that puromycin incorporation at 20 mM glucose plateaued from 2 h of treatment (Fig. 2*D*), and we selected 4 h glucose pulse for the following experiments. Note that, in this setting, cycloheximide pretreatment blocked puromycin incorporation in all tested conditions (Fig. 2*D*). Based on this result, we concluded that the SUnSET technique is a valid method for measuring *in vitro* changes in protein translation in human β-cells.

### Glucose increases translation of the proconvertase PCSK1 in EndoC-βH2 cells

**Figure 3:**
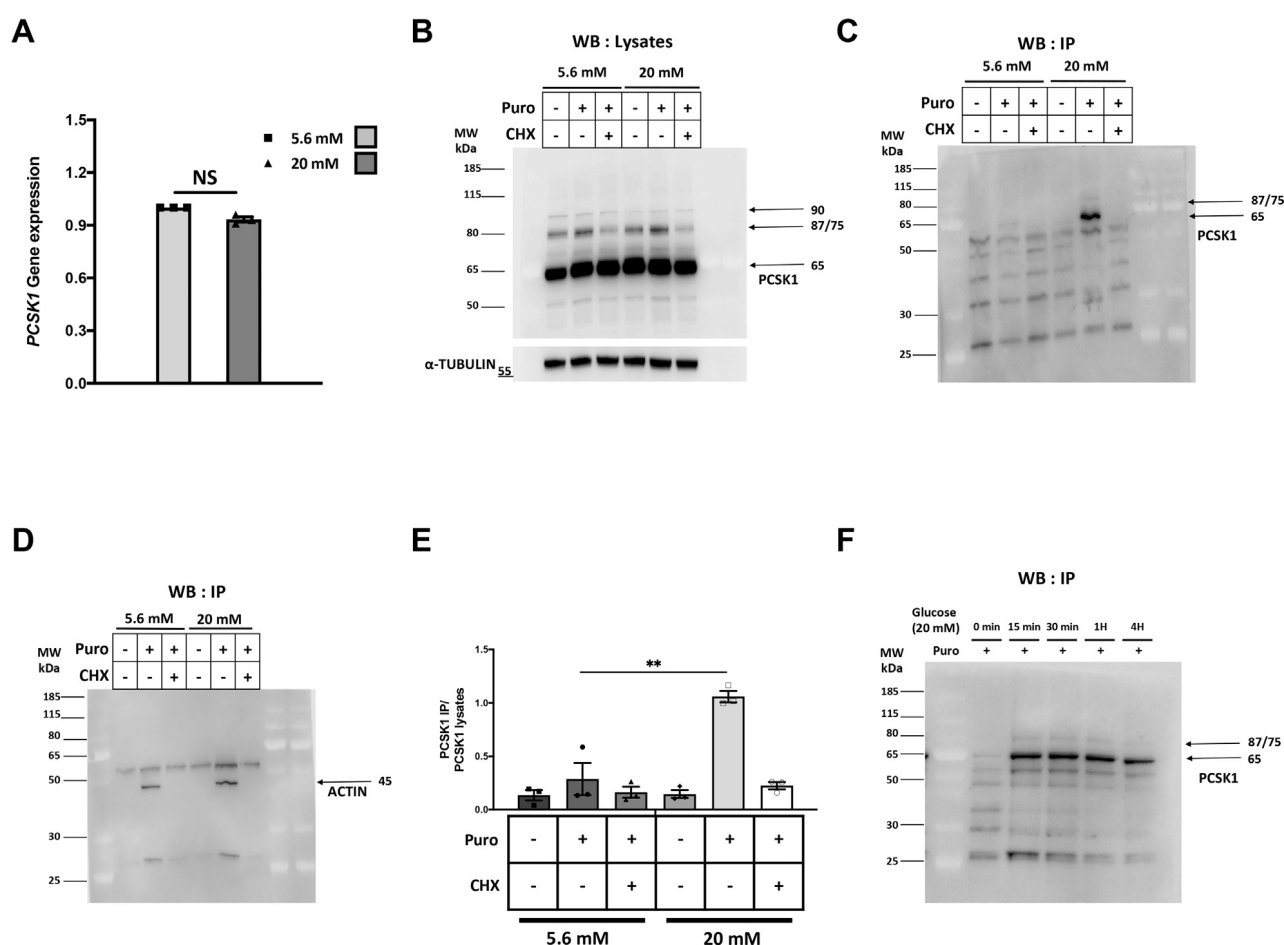
**Glucose induces PCSK1 translation in EndoC-βH2 cells.** EndoC-βH2 cells were cultured at 5.6 mM glucose. Glucose concentration was next increased to 20 mM for 4 h with puromycin added during the last 10 min. *A*, RT-qPCR indicating that glucose does not modulate PCSK1 mRNA levels in EndoC-βH2 cells. The error bars represent the mean ± SEM of three separate experiments. *B*, detection of PCSK1 by Western blot in total protein lysates. *C* and *D*, proteins were immunoprecipitated using an anti-puromycin antibody, run on a gel, and blotted using either an anti-PCSK1 antibody (*C*) or an anti-actin antibody (*D*). *E*, quantification of puromycin incorporation in PCSK1 relative to total PCSK1. The error bars represent the mean ± SEM of three separate experiments. ∗∗*p* < 0.01. *F*, EndoC-βH2 cells were cultured at 5.6 mM glucose. Glucose concentration was next increased to 20 mM for 15 min to 4 h with puromycin added during the last 10 min. Proteins were immunoprecipitated using an anti-puromycin antibody, run on a gel, and blotted using an anti-PCSK1 antibody.

PCSK1, also called PC1/3, is a proconvertase expressed by β-cells and necessary for the processing of proinsulin into insulin (25, 26, 27, 28). In mouse islets, PCSK1 expression has been shown to be regulated by glucose at the translational levels (6). We thus looked at the regulation of PCSK1 expression by glucose in EndoC-βH2 cells. First, we observed that, in these cells, glucose did not increase PCSK1 mRNA steady-state levels (Fig. 3*A* and (9)). Moreover, Western blot analysis indicated that, upon glucose stimulation, variation in PCSK1 protein steady-state levels could not be observed on total lysates from EndoC-βH2 cells (Fig. 3*B* and Fig. S2 for quantification), which might be due to the stability of the protein. We thus used the more dynamic SUnSET approach. We stimulated EndoC-βH2 cells with 20 mM glucose for 4 h and pulsed them for 10 min with puromycin. Immunoprecipitations were performed with anti-puromycin antibodies, followed by Western blot using anti-PCSK1 antibody. It demonstrated that 20 mM glucose increased puromycin incorporation into PCSK1, which was not the case when EndoC-βH2 cells were pretreated with cycloheximide or when puromycin was omitted (Fig. 3, *C* and *E* for quantification). As expected, in EndoC-βH2 cells, glucose did not modulate actin translation (Fig. 3*D*). Of interest, PCSK1 neosynthesis upon glucose stimulation was fast as a time course experiment indicated that a 15-min glucose treatment was sufficient to induce PCSK1 translation (Fig. 3*F*). Taken together, we validated the SUnSET method as an accurate approach to identify proteins whose translation is modulated by glucose in EndoC-βH2 cells.

### Identification by mass spectrometry of neosynthesized proteins regulated by glucose in EndoC-βH2 cells

**Figure 4:**
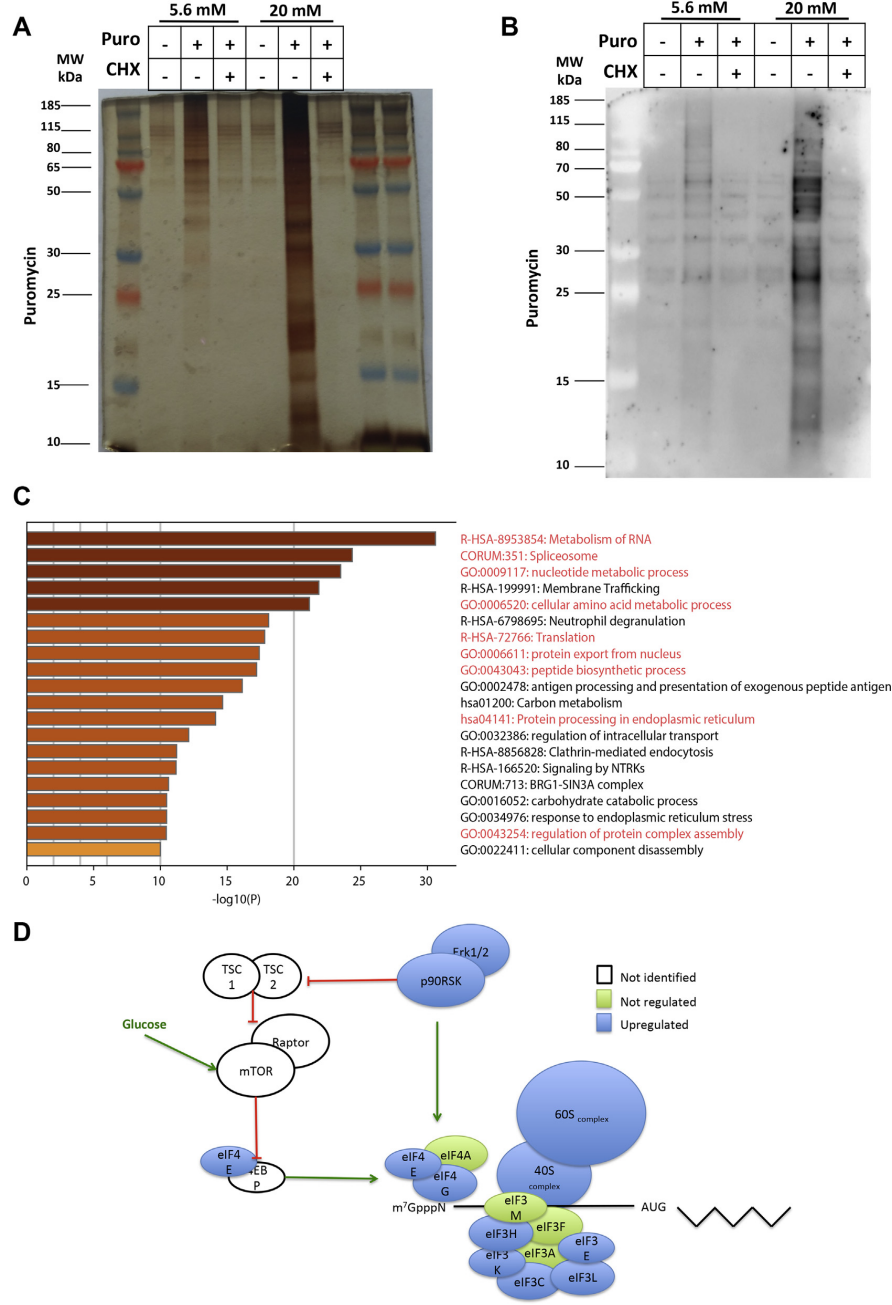
**Identification of newly translated proteins upon glucose treatment in EndoC-βH2 cells.** EndoC-βH2 cells were cultured at 5.6 mM glucose. Glucose concentration was next increased to 20 mM for 4 h with puromycin added during the last 10 min. In some cases, EndoC-βH2 cells were treated for 1 h with 50 μg/ml CHX before glucose pulse. Proteins were immunoprecipitated with an anti-puromycin antibody and run on gel Bolt 12% Tris-Bis. *A*, total proteins were visualized by silver staining. *B*, proteins that had incorporated puromycin were detected following incubation with an anti-puromycin antibody. *C*, Gene Ontology (GO) annotation and Gene Set Enrichment Analysis (GSEA) of the differentially expressed proteins using Metascape analysis highlighting interconnected protein networks involved after translation improved by high glucose in EndoC-βH2 cells. *D*, scheme showing the neonascent peptides involved in the translation initiation complex modulated by glucose and identified in two independent mass spectrometry experiments (upregulated proteins in *blue*, not-regulated proteins in *green*).

To identify additional human β-cell proteins with increased translation under high-glucose condition, we performed a 4-h pulse with 20 mM glucose including a 10-min puromycin treatment followed by immunoprecipitation using anti-puromycin antibodies. Protein silver staining indicated that glucose increased the total amount of immunoprecipitated proteins (Fig. 4*A*). Note that extremely limited amounts of proteins were immunoprecipitated when puromycin was omitted or when EndoC-βH2 cells were pretreated with cycloheximide (Fig. 4*A*). Similar conclusions were obtained when proteins that had incorporated puromycin were immunoprecipitated and blotted using puromycin antibodies: again, the puromycin signal was higher in EndoC-βH2 cells cultured at 20 *versus* 5.6 mM glucose and this difference was blunted when cells were pretreated with cycloheximide (Fig. 4*B*).

Immunoprecipitated proteins were digested with trypsin and analyzed by mass spectrometry by the S-TRAP method. Data were processed with MaxQuant (29). We performed two independent experiments and focused on proteins common to both experiments. We identified more than 2000 proteins by mass spectrometry. We detected 703 proteins that included PCSK1 with a 2-fold enrichment in the 20 mM glucose-treated groups compared with 5.6 mM. Such proteins were not detected in controls performed in the absence of puromycin treatment or upon cycloheximide pretreatment (Table S1). Gene Set Enrichment Analysis using Metascape (30) highlighted proteins translated upon glucose stimulation associated with metabolism of RNA, RNA splicing, amino acid metabolism, and protein translation (Fig. 4*C*). As an example, glucose induced the translation of many proteins associated with the eukaryotic ribosomal subunits 40S and 60S (Fig. 4*D*). We also observed an increased translation of most of the eukaryotic Initiation Factor 4 (eIF4) protein family members (eIF4E/G/H) involved in recognition and binding of the 5' cap structure of mRNA. Finally, the translation of proteins of the eukaryotic Initiation Factor 3 (eIF3) complex (eIF3 B, C, E, H, K, and L) that leads to the assembly of the 40S ribosomal subunit on mRNA in the translation initiation complex was also controlled by glucose. We also observed an increased translation of seven aminoacyl-tRNA synthetase (amino acid code: H, K, W, F, M, S, Q, and Y) linking tRNA with their respective amino acids. Such data thus further support the fact that glucose improves protein translation in human β-cells.

**Figure 5:**
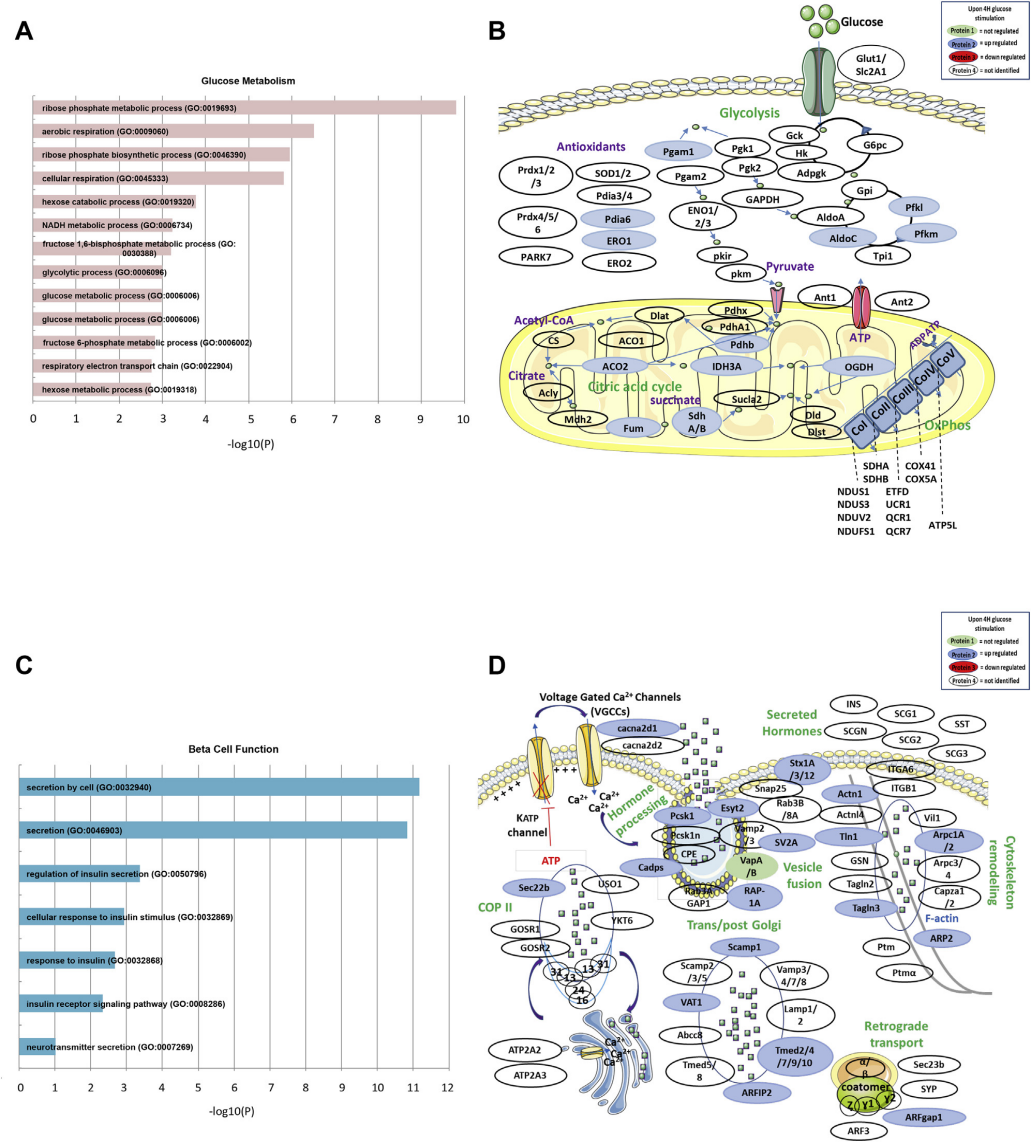
**Glucose effect on translation of proteins involved in glucose metabolism and β-cell function.***A*, glucose metabolism–linked Gene Ontology (GO) analysis of islet proteins differentially translated with glucose. *B*, scheme showing the neonascent proteins modulated by glucose involved in glucose metabolism (upregulated proteins in *blue* and not-regulated proteins in *green*). *C*, β-cell function mechanism–associated GO analysis of islet proteins differentially translated with glucose. *D*, scheme showing the neonascent proteins modulated by glucose involved in β-cell function (upregulated proteins in *blue* and not-regulated proteins in *green*).

By performing targeted Gene Ontology annotation using the PANTHER classification, we highlighted many proteins involved in glucose metabolism (Fig. 5*A*). This was the case for proteins associated with glycolysis such as 6-phosphofructokinase, liver type (PFKL) and muscle type (PFKM), and Aldolase C (ALDOC). This was also the case for enzymes of the tricarboxylic acid (TCA) cycle such as pyruvate dehydrogenase E1 component subunit beta (PDHB) and isocitrate dehydrogenase subunit alpha (IDH3A). This was finally the case for the five complexes (CoI-CoV) involved in oxidative phosphorylation. As examples, it was the case for NADH dehydrogenase ubiquinone iron-sulfur proteins 1 and 3 (NDUS1; NDUS3), NADH dehydrogenase ubiquinone flavoprotein 2 (NDUV2), and NADH-ubiquinone oxidoreductase 75 kDa subunit 1 (NDUFS1) from complex I (CoI), and the succinate dehydrogenase ubiquinone flavoprotein subunit proteins A and B (SDHA, SDHB) from complex II (CoII) (Fig. 5*B*).

The PANTHER classification also highlighted a large number of proteins implicated in insulin exocytosis (Fig. 5*C*). As examples, we further confirmed increased puromycin incorporation upon glucose treatment in PCSK1 as observed by Western blot (Fig. 3*C*). We also found that glucose induced translation of Ras-like small G protein RAP-1A that is required for the first phase of potentiation of insulin secretion (31). We also detected proteins such as calcium-dependent secretion activator 1 (CADPS) known to be implicated in the calcium signaling pathway, in large dense-core vesicles formation and important to insulin secretion (Fig. 5*D*) (32). We also identified the voltage-dependent calcium channel subunit alpha-2/delta-1 (CACNA2D1) that is known to affect insulin secretion by suppressing voltage-gated calcium channel transport to the plasma membrane, thereby reducing Ca^2+^ signaling (33). CACNA2D1 acts on endosomes linked to the Golgi as a recycling carrier to the cell surface such as the glucose translated secretory carrier-associated membrane protein 1 (SCAMP1) (Fig. 5*D*). Finally, glucose increased the translation of key proteins involved in insulin exocytosis. This was the case for proteins important for vesicle fusion (SNARE proteins) such as syntaxin-1A (STX1A), syntaxin-3 (STX3), and syntaxin-12 (STX12) and for multiple proteins involved in vesicular protein trafficking such as member of the transmembrane P24 trafficking protein (TMED7, TMED9, TMED10) and the vesicle-trafficking protein SEC22b (SC22B) (Fig. 5*D*).

### MAPK and PRKAR2A translation is modulated by glucose in EndoC-βH2 cells

**Figure 6:**
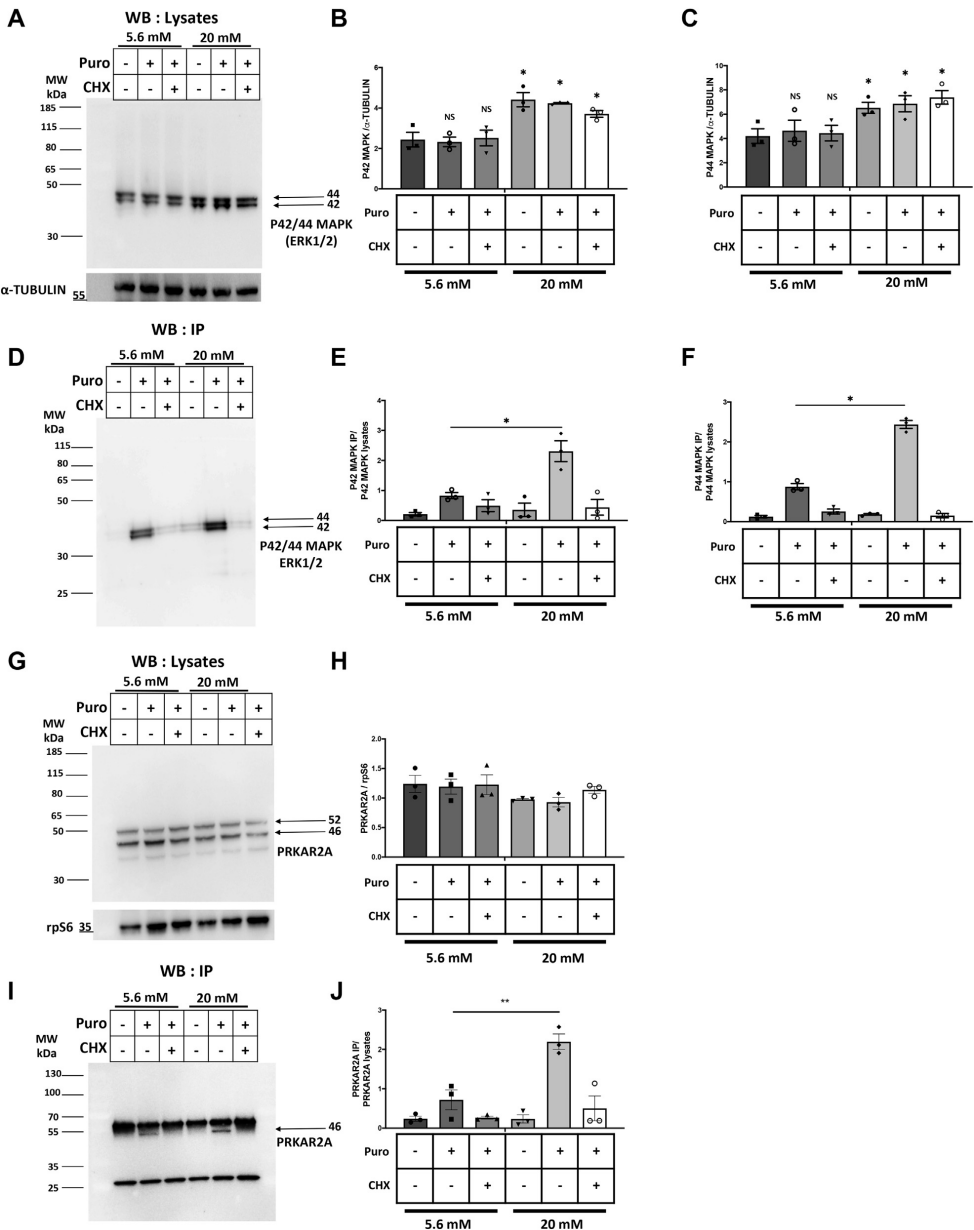
**Glucose induces MAPK and PRKAR2A translation in EndoC-βH2 cells.** EndoC-βH2 cells were cultured at 5.6 mM glucose. Glucose concentration was next increased to 20 mM for 4 h with puromycin added during the last 10 min. *A*, detection of MAPK by Western blot in total protein lysates. *B* and *C*, quantification of MAPK proteins relative to α-TUBULIN. *D*, proteins were immunoprecipitated using an anti-puromycin antibody, run on a gel, and blotted using an anti-P42/44MAPK antibody. *E* and *F*, quantification of puromycin incorporation in MAPK proteins relative to total MAPK. *G*, detection of PRKAR2A by Western blot in total protein lysates. *H*, quantification of total PRKAR2A relative to rpS6 as a depot control protein. *I*, proteins were immunoprecipitated using an anti-puromycin antibody, run on a gel, and blotted using an anti-PRKAR2A antibody. *J*, quantification of puromycin incorporation in PRKAR2A relative to total PRKAR2A. The error bars represent the mean ± SEM of three separate experiments. NS, not significant, ∗*p* < 0.05; ∗∗*p* < 0.01.

Among the 703 proteins showing a 2-fold enrichment of puromycin incorporation under 20 mM glucose in EndoC-βH2 cells (Table S1), we selected three proteins for further validation. We first focused on mitogen-activated protein kinase 1 (MAPK1) (also called Erk1 or p42MAPK) and MAPK3 (also called Erk2 or p44MAPK). MAPK signaling is ubiquitous and involved in many cellular functions including cell growth, survival, and differentiation. In β-cells, MAPKs are required for β-cell mass regulation and insulin secretion (34). Western blot analysis indicated that glucose increased MAPK levels (Fig. 6, *A*–*C* for quantification). Immunoprecipitations using anti-puromycin antibody followed by Western blot using anti-MAPK antibody demonstrated a parallel increase of puromycin incorporation into MAPKs (Fig. 6, *D*–*F* for quantification).

We next concentrated on the cAMP-dependent protein kinase type II-alpha regulatory subunit 1 (PRKAR2A). This protein is one of the four different regulatory subunits of the cAMP-dependent protein kinase A (PKA). The inactive holoenzyme of PKA is a tetramer composed of two catalytic and two regulatory subunits as PRKAR2A. PKA signaling is essential for β-cell differentiation and insulin secretion (35, 36). Here, Western blot analysis indicated that glucose did not regulate the PRKAR2A steady-state level (Fig. 6, *G* and *H* for quantification). On the other hand, by using the dynamic SUnSET approach we observed that glucose induces PRKAR2A translation (Fig. 6, *I* and *J* for quantification).

Taken together, through the use of a puromycin incorporation-based approach, we demonstrated that glucose regulates the translation of proteins involved in glucose metabolism and insulin exocytosis in human β-cells.

## Discussion

Glucose has been shown to play major roles in β-cell proliferation, survival, and function both in rodent and human (37, 38, 39). However, we recently observed that, although glucose regulates the transcription of many genes in rodent β cells, such an effect is limited in human (9). We reasoned that glucose might act at the posttranscriptional level to regulate protein synthesis in human β-cells. Here we demonstrate that it is indeed the case. By using the nonradioactive SUnSET assay linked to mass spectrometry, we demonstrate that glucose upregulates not only the basal translation machinery but also the translation of metabolic proteins associated with increased glycolysis, production of TCA metabolites, oxidative phosphorylation, and insulin machinery, all described to regulate glucose-stimulated insulin secretion. Therefore, we conclude that glucose controls protein levels at the translational step in human pancreatic β-cells.

To investigate the involvement of glucose in protein translation, we treated the human β-cell line EndoC-βH2 with glucose, which was followed by a pulse with puromycin, an aminonucleoside antibiotic produced by *Streptomyces alboniger*, which incorporates into elongating peptide chains (40). It was previously observed by using ^32^P labeled puromycin that a low concentration of puromycin does not interfere with the overall rate of protein synthesis and incorporates only at the C terminus of full-length protein (12). In this study, the authors propose a possible model to explain the specific binding of puromycin to the C terminus of full-length protein. Puromycin at high concentrations competes with aminoacyl-tRNA, causing premature termination of protein synthesis and forming peptidyl puromycin by being linked to the growing peptide chain. On the other hand, puromycin at lower concentrations used in SUnSET hardly inhibits protein synthesis, so that it could bond specifically only at the stop codon of full-length proteins where puromycin does not compete anymore with aminoacyl-tRNA. Finally, to the best of our knowledge, no study has described that certain proteins are more sensitive or resistant to C-terminal puromycin labeling than others. Based on this observation, the SUnSET approach is now recognized as an efficient alternative to protein synthesis measurements performed using ^35^S-methionine (10). SUnSET is then used as a simple inexpensive nonradioactive technique that does not require methionine or other amino acid depletion prior to labeling (10, 14–18). By using this approach, we identified more than 2000 proteins expressed by human β-cells. Here, we used SUnSET to analyze the effects of glucose on the nascent proteome in human β-cells. A recent study criticized the SUnSET approach that might interfere with the mRNA translation machinery when cells are cultured under sharp starved conditions (41). We thus did not starve EndoC-βH2 cells but instead, analyzed the neonascent proteome by comparing normoglycemic level of 5.6 mM glucose with higher glucose concentrations. Here, data were obtained using the human β-cell line EndoC-βH2 (19), and it will be interesting to determine whether glucose regulates the translation of similar proteins in human primary β-cells. However, the use of SUnSET approach with primary β-cells might be difficult for two reasons: i) Human islet preparations contain not only β-cells but also alpha, delta, and PP cells and are also always contaminated with acinar and duct cells, the frequency of each cell type being extremely variable from one preparation to the other. A β-cell purification step will thus be necessary. ii) Only limited amounts of primary human β-cells can currently be obtained, which are incompatible with the large amounts of cells necessary in the SUnSET protocol that will need to be miniaturized before its use.

Proinsulin processing in β-cells is a tightly regulated process, and defective proinsulin processing has been implicated in the pathogenesis of both type 1 and type 2 diabetes (42, 43). In rodent β-cells both PCSK1 and its paralog PCSK2 are necessary for proinsulin processing (6). In human, PCSK1 is necessary for proinsulin processing, and patients with mutant *PCSK1* have increased circulating proinsulin levels (26). PCSK1 also seems sufficient in a human context based on a recent study that brought evidences indicating that human β-cells process proinsulin through PCSK1 without a need of PCSK2 (44). This can be added to the list of differences between rodent and human β-cells (45). We report here that glucose increases the translation of the proconvertase PCSK1 in human β-cells, which is similar to what has been described for rodent β-cells (6). Of interest, we observed that glucose specifically increased the translation of PCSK1, without any detectable effect on PCSK2.

Our data indicate that glucose increased the translation of many enzymes involved in the TCA cycle. Short-term glucose treatments might thus increase β-cell differentiation to improve the function of β-cell. This hypothesis correlates with recent results indicating that circadian glucose pulses induce β-cell differentiation and maturation of human-stem-cell-derived islet (39). It is also interesting to observe that most TCA substrates produced under high-glucose treatment are also known to be involved in chromatin modification and DNA methylation. Specifically, histone acetylation is dependent on the availability of acetyl-CoA, which provides the necessary acetyl groups to enable the reaction. As another example, α-ketoglutarate is an essential cofactor of histone demethylases (46). Finally, we could then suggest that glucose increases the translation of TCA cycle enzymes that improve the maturity of human β-cells. Taken together, such studies performed on human β-cell lines complement previous studies on the regulation of translation by glucose in whole rodent islets.

Until now, the regulation by glucose of protein steady-states levels in human pancreatic β-cells has been mainly studied through mRNA expression analyses (4, 9, 47). Here, by applying to human β-cells the SUnSET approach coupled to mass spectrometry, we have uncovered a list of proteins whose translation is glucose sensitive. We demonstrate that, although glucose has limited effects at the transcriptional level in human β-cells (9), it is a key regulator of protein translation. The SUnSET assay can be used (through FACS or Western blot or coupled to mass spectrometry) as a simple alternative to traditional methods of protein synthesis measurements in human β-cells. This method can now be used to evaluate the regulation at the translational level under several treatments and to discover β-cell-specific translation activators.

## Experimental procedures

### Culture of human EndoC-βH2 cell line

EndoC-βH2 cells were cultured in Dulbecco’s modified Eagle’s medium (5.6 mM glucose) (Gibco) with 2% BSA fraction V, fatty acid free (Roche), 50 μM β-mercaptoethanol (Sigma-Aldrich), 10 mM nicotinamide (Calbiochem), 5.5 μg/ml transferrin (Sigma-Aldrich), 6.7 ng/ml sodium selenite (Sigma-Aldrich), 100 units/ml Penicillin, and 100 mg/ml Streptomycin (Thermo Fischer Scientific) as described (8(19)). Cells were seeded at a 50% confluence on plates coated with Matrigel (1.2%; Sigma-Aldrich) and fibronectin (3 μg/ml; Sigma-Aldrich), cultured at 37 °C in 5% CO_2_, and passaged once a week. For all experiments, cells were plated 48 h before treatment.

### Surface sensing of translation technique

EndoC-βH2 cells (either glucose starved at 0.5 mM for 24 h or cultured at 5.6 mM glucose) were treated with different concentrations of glucose. They were pulsed during the last 10 min with 10 μg/ml of puromycin (Thermo Fischer Scientific) and finally washed three times with Dulbecco's phosphate-buffered saline (Gibco). In some experiments, cells were pretreated for 1 h with either 50 nM rapamycin or 50 μg/ml cycloheximide (both from Sigma-Aldrich).

### Immunoblotting

EndoC-βH2 cells were lysed in RIPA buffer (Sigma-Aldrich). Protein concentrations were quantified by Pierce BCA protein assay kit (Thermo Fischer Scientific). Proteins were resolved by SDS-PAGE and transferred to a membrane using the iBlot2 Gel transfer device (Thermo Fischer Scientific). Western blots were performed using the following primary antibodies diluted (1/1000) in TBS 3% BSA 0.1% Tween-20: phospho-rpS6 (Ser240/244: D68F8), total rpS6 (5G10), 4EBP1 (53H11), and p44/42 MAPK (Erk1/2) (9102S) (all from Cell Signaling); anti-puromycin (MABE343-12D10) and anti-Prohormone convertase PCSK1 (AB10553) (both from Merck Millipore); anti-PRKAR2A (612243) from (BD Biosciences). α-TUBULIN (T9026) and β-ACTIN (A5441) (both from Sigma-Aldrich) were diluted (1/2000) in TBS 5% milk 0.1% Tween-20. After washing, membranes were incubated with species-specific horseradish peroxidase–linked secondary antibodies (Cell Signaling) diluted (1/2000–1/10,000) in TBS 1% milk 0.1% Tween-20 and visualized on an ImageQuant LAS 4000 following ECL exposure (GE Healthcare). Densitometric quantification of Western blots was done using ImageJ software and normalized to α-tubulin expression. Immunoblotting experiments were performed three times.

### Immunoprecipitation

Cell lysates were prepared as for Western blot and incubated overnight with anti-puromycin antibody (1 μg/200 μg protein lysate) in lysis buffer on a rotary wheel at 4 °C. Sufficient quantity of protein A/G agarose beads (Thermo Fischer Scientific) was washed overnight in lysis buffer on a rotary wheel at 4 °C. The next day, the beads were added on each sample and the complexes were mixed for 1 h. Beads were recovered by centrifugation and washed with lysis buffer to eliminate nonspecific bonds. They were resuspended in Laemmli buffer containing β-mercaptoethanol and heated at 95 °C for 5 min for elution. Western blot and mass spectrometry were next performed. Gels were stained using SilverQuest Silver staining Kit (Thermo Fischer Scientific) as per the instruction manual. IP experiments were performed three times.

### Fluorescence-activated cell sorting

EndoC-βH2 cells were treated with different concentrations of glucose and pulsed with puromycin as described above. They were collected using trypsin, fixed, and permeabilized using Fixation/permeabilization solution kit (BD Biosciences) for 1 h at 4 °C under agitation before centrifugation. Cells were next incubated with anti-puro-AF488 (MABE343-AF488) (Merck Millipore) diluted (1/1000) in Permwash (BD Biosciences) under agitation at 4 °C for 3 h. Cells were next washed with Permwash (1X) and resuspended in Dulbecco's phosphate-buffered saline (1X) before flow cytometric acquisition (FACS Aria III, Becton Dickinson). Data were analyzed using FlowJo software.

### RNA isolation, reverse transcription, and quantitative PCR

Total RNA of EndoC-βH2 cells was isolated and used to synthesize cDNA as described (8, 9). Quantitative RT-qPCR was performed using Power SYBR Green mix (Applied Biosystems) with a QuantStudio analyzer (Thermo Fisher Scientific). Custom primers were designed with NCBI Primer-Blast (48), and their efficiency was determined following serial dilutions of cDNA samples. PCSK1 primer sequences: Fw (5’-3’): GATCGTGTGATATGGGCTGAA; Rv (5’-3’): TCCACATGGGATCATTGAAGAG. Cyclophilin-A primer sequences used for normalization Fw (5’-3’): ATGGCAAATGCTGGACCCAACA; Rv (5’-3’): ACATGCTTGCCATCCAACCACT.

### Mass spectrometry protein identification and quantification

S-Trap micro spin column (Protifi) digestion was performed on IP eluates in Laemmli buffer according to manufacturer’s protocol, using four washing steps for thorough SDS elimination. Samples were digested with 2 μg of trypsin (Promega) at 47 °C for 90 min. After elution, peptides were vacuum dried and resuspended in 35 μl of 10% acetonitrile, 0.1% trifluoroacetic acid (TFA) in HPLC-grade water prior to MS analysis. For each run, 5 μl was injected in a nanoRSLC-Q Exactive PLUS (RSLC Ultimate 3000) (Thermo Scientific). Peptides were loaded onto a μ-precolumn (Acclaim PepMap 100 C18, cartridge, 300 μm i.d. × 5 mm, 5 μm) (Thermo Scientific) and were separated on a 50-cm reversed-phase liquid chromatographic column (0.075 mm ID, Acclaim PepMap 100, C18, 2 μm) (Thermo Scientific). Chromatography solvents were (A) 0.1% formic acid in water and (B) 80% acetonitrile, 0.08% formic acid. Peptides were eluted from the column with the following gradient: 5% to 40% B (120 min), 40% to 80% (1 min). At 121 min, the gradient stayed at 80% for 5 min and, at 126 min, it returned to 5% to re-equilibrate the column for 20 min before the next injection. One blank was run between each sample to prevent sample carryover. Peptides eluting from the column were analyzed by data-dependent MS/MS, using top-10 acquisition method. Peptides were fragmented using higher-energy collisional dissociation. Briefly, the instrument settings were as follows: resolution was set to 70,000 for MS scans and 17,500 for the data-dependent MS/MS scans in order to increase speed. The MS AGC target was set to 3 × 10^6^ counts with maximum injection time set to 200 ms, while MS/MS AGC target was set to 1 × 10^5^ with maximum injection time set to 120 ms. The MS scan range was from 400 to 2000 *m/z*.

### Data processing after MS acquisition

The MS raw files were processed with Proteome Discoverer software version 1.4.0.288 and searched using Mascot search engine version 2.2 against the database of *Homo sapiens* from Swiss-Prot 04/2019 (20,415 entries). To search parent mass and fragment ions, we set an initial mass tolerance of 5 ppm and 0.05 Da, respectively. The minimum peptide length was set to six amino acids. A fixed-value PSM validation was set based on Peptide Mascot (Ionscore) threshold at 25. Semi-trypsin cleavage was set, allowing up to two missed cleavage sites. Carbamidomethylation (Cys) was set as fixed modification, whereas oxidation (Met), N-term acetylation and puromycin (C-term) were set as variable modifications. We performed two independent experiments. Only proteins identified with at least two peptides were retained. Proteins identified in both experiments were further studied. Proteins identified in negative controls performed in the absence of puromycin (-puro) were excluded from the analysis. Subsequently, the ratio for the most important sample (+puro at 20 mM) was calculated compared with the control (+puro 5.6 mM) for each experiment. We focused only on proteins with 2-fold enrichment in the 20 mM glucose-treated groups compared with 5.6 mM. The complete mass spectrometry proteomics data have been deposited to the ProteomeXchange Consortium *via* the PRIDE partner repository with the dataset identifier PXD023996.

Gene Set Enrichment Analysis of differentially translated proteins was performed using Metascape (30) and Protein ANalysis THrough Evolutionary Relationship (PANTHER) software (http://www.pantherdb.org) (49).

### Statistical analysis

Data were analyzed by GraphPad Prism 8 software and were presented as the mean ± SEM. For comparison between two mean values, statistical significance was estimated using Student’s *t* test. For comparison among three values, one-way ANOVA was used with Holm–Sidak's multiple comparisons test.

## Data availability

The datasets generated and/or analyzed during the current study are available from the corresponding authors. The complete mass spectrometry proteomics data have been deposited to the ProteomeXchange Consortium *via* the PRIDE partner repository with the dataset identifier PXD023996 (https://www.ebi.ac.uk/pride/archive/projects/PXD023996/private).

## Supporting information

This article contains supporting information.

## Conflict of interest

The authors declare that they have no conflicts of interest with the contents of this article.
