# Supplementary material for: Glucose treatment of human pancreatic β-cells enhances translation of mRNAs involved in energetics and insulin secretion
Source: J Biol Chem. 2021 May 27;297(1):100839. doi: 10.1016/j.jbc.2021.100839 (PMC8253965; doi:10.1016/j.jbc.2021.100839)
Supplement: Supplemental Figures S1 and S2 [file mmc1.pdf]

Table. S1. Identification of specific puromycin-labeled nascent proteome induced by glucose in EndoC-βH2 cells.

Summary of 703 enriched proteins ≥ 2 fold after stimulation with 20 mM glucose for 4H and then 10 minutes with 10µg/mL puromycin in EndoC-βH2 cells. EndoC-βH2 cells were cultured at 5.6mM glucose. Glucose concentration was next increased to 20mM for 4h with puromycin added during the last 10min. 703 identified proteins (703) are induced under 20mM glucose more than 2 fold (≥ 2 fold).

Fig. S1. Glucose regulates mTOR activity in EndoC-βH2 cells.

EndoC-βH2 cells were treated without or with rapamycin (RAPA: 50nM) for 1h followed by a 30 min glucose pulses. **(A)**. Quantification of P-rpS6 phosphorylation relative to total rpS6. **(B)** Quantification of 4E-BP1 phosphorylation relative to total α−TUBULIN. The error bars represent the mean ± SEM of three separate experiments. *P<0.05, **P<0.01, ***P<0.005 and ****P<0.001.

Fig. S2. Glucose induces PCSK1 translation in EndoC-βH2 cells.

EndoC-βH2 cells were cultured at 5.6mM glucose. Glucose concentration was next increased to 20mM for 4h with puromycin added during the last 10min. Detection of PCSK1 by Western blot in total protein lysates was quantified relative to total α−TUBULIN. The error bars represent the mean ± SEM of three separate experiments. NS=Not Significant.

Figure S1

A

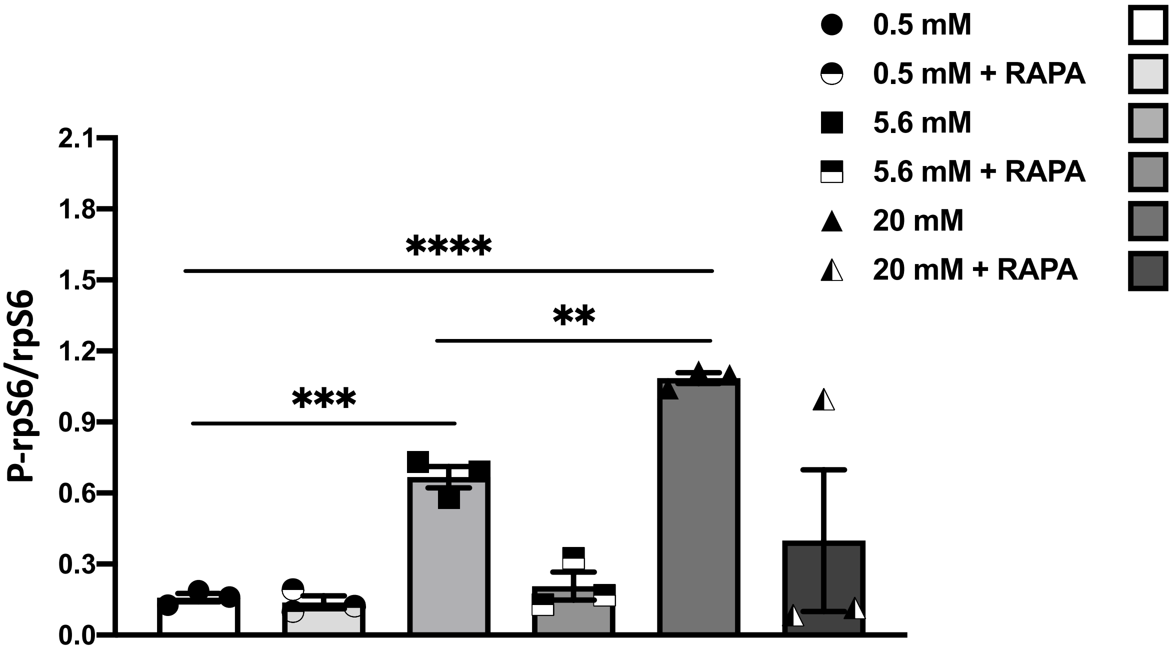

0.5 mM
0.5 mM + RAPA
5.6 mM
5.6 mM + RAPA
20 mM
20 mM + RAPA
P-rpS6/rpS6
2.1
1.8
1.5
1.2
0.9
0.6
0.3
0.0
****
**
***

B

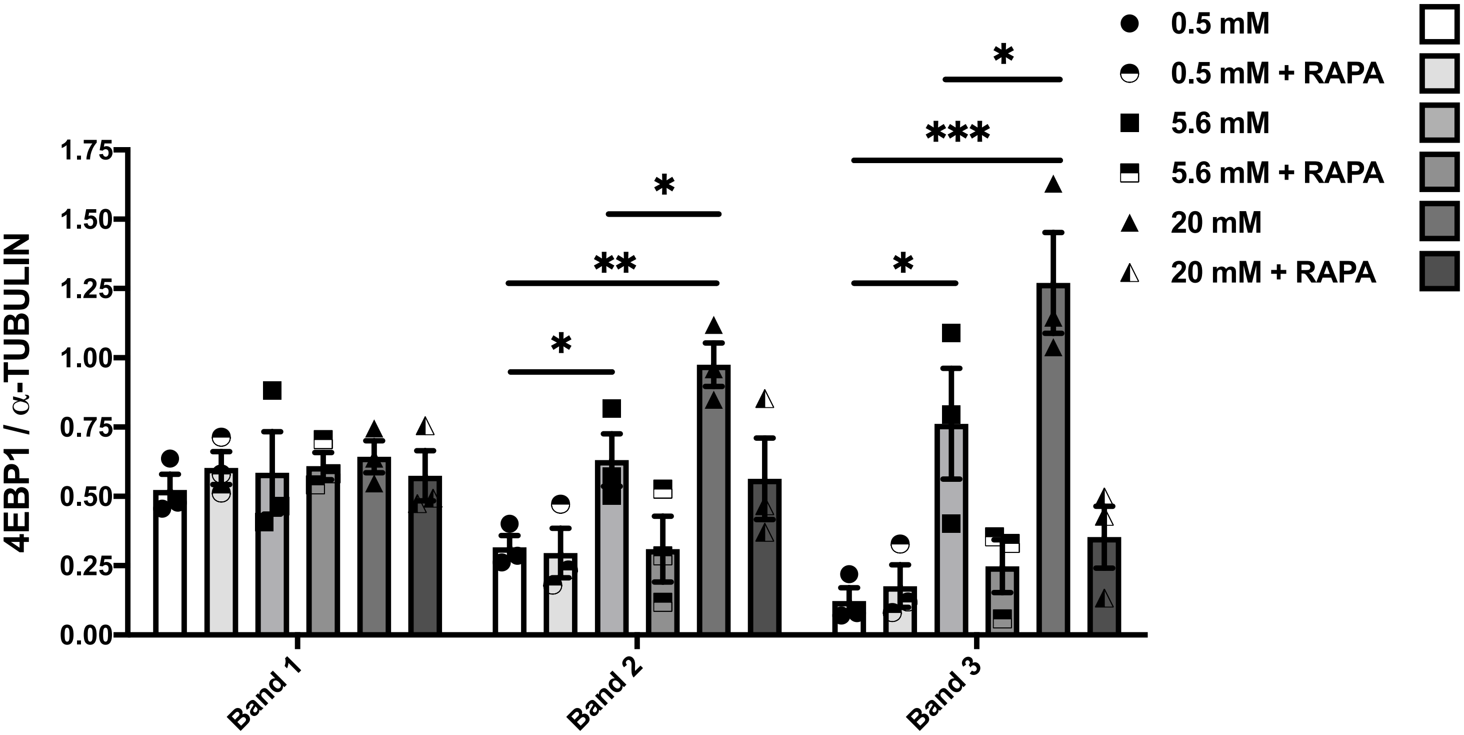

0.5 mM
0.5 mM + RAPA
5.6 mM
5.6 mM + RAPA
20 mM
20 mM + RAPA
4EBP1 / α-TUBULIN
1.75
1.50
1.25
1.00
0.75
0.50
0.25
0.00
Band 1
Band 2
Band 3
*
***
**

Figure S2

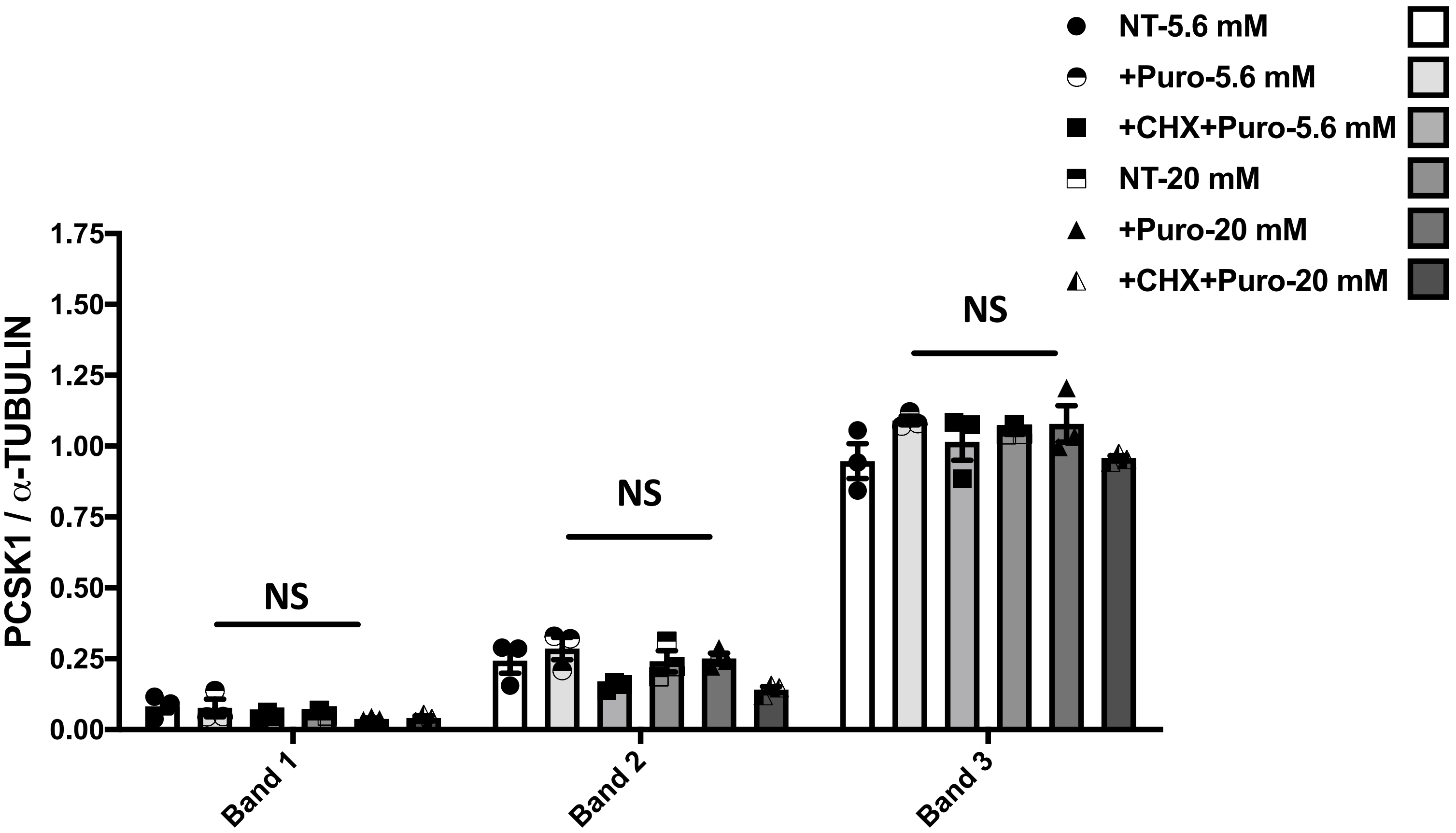

NT-5.6 mM
+Puro-5.6 mM
+CHX+Puro-5.6 mM
NT-20 mM
+Puro-20 mM
+CHX+Puro-20 mM
PCSK1 / α-TUBULIN
1.75
1.50
1.25
1.00
0.75
0.50
0.25
0.00
NS
NS
NS
Band 1
Band 2
Band 3
